# Facial expression recognition as a candidate marker for autism spectrum disorder: how frequent and severe are deficits?

**DOI:** 10.1186/s13229-018-0187-7

**Published:** 2018-01-30

**Authors:** E. Loth, L. Garrido, J. Ahmad, E. Watson, A. Duff, B. Duchaine

**Affiliations:** 1 Sackler Institute for Translational Neurodevelopment, Institute of Psychiatry, Psychology and Neuroscience, King’s College London, De Crespigny Park, Denmark Hill, London, SE5 8AF UK; 2 Department of Forensic and Neurodevelopmental Sciences, Institute of Psychiatry, Psychology and Neuroscience, King’s College London, London, UK; 3 Division of Psychology, Department of Life Sciences, Brunel University, London, UK; 4 Department of Psychological and Brain Sciences, Dartmouth College, Hanover, USA

**Keywords:** Autism Spectrum disorder, Facial expression recognition, Biomarker

## Abstract

**Background:**

Impairments in social communication are a core feature of Autism Spectrum Disorder (ASD). Because the ability to infer other people’s emotions from their facial expressions is critical for many aspects of social communication, deficits in expression recognition are a plausible candidate marker for ASD. However, previous studies on facial expression recognition produced mixed results, which may be due to differences in the sensitivity of the many tests used and/or the heterogeneity among individuals with ASD. To ascertain whether expression recognition may serve as a *diagnostic marker* (which distinguishes people with ASD from a comparison group) or a *stratification marker* (which helps to divide ASD into more homogeneous subgroups), a crucial first step is to move beyond identification of mean group differences and to better understand the frequency and severity of impairments.

**Methods:**

This study tested 46 individuals with ASD and 52 age- and IQ-matched typically developing (TD) participants on the Films Expression Task, which combines three key features of real-life expression recognition: naturalistic facial expressions, a broad range of simple and complex emotions, and short presentation time. Test-retest reliability was assessed in 28 individuals who did not participate in the main study and revealed acceptable reliability (ICC *r* = .74).

**Results:**

Case-control comparisons showed highly significant mean group differences for accuracy (*p* = 1.1 × 10^− 10^), with an effect size (Cohen’s *d* = 1.6), more than twice as large as the mean effect size reported in a previous meta-analysis (Uljarevic and Hamilton, 2012, *J Autism Dev Disord*). The ASD group also had significantly increased mean reaction times overall (*p* = .00015, *d* = .83) and on correct trials (*p* = .0002, *d* = .78). However, whereas 63% of people with ASD showed severe deficits (they performed below two standard deviations of the TD mean, a small subgroup (15.3%) performed normally (within one standard deviation of the mean).

**Conclusion:**

These findings indicate that the majority of people with ASD have severe expression recognition deficits and that the Films Expression Test is a sensitive task for biomarker research in ASD. Future work is needed to establish whether ASD subgroups with and without expression recognition deficits differ from one another in terms of their symptom profile or neurobiological underpinnings.

**Electronic supplementary material:**

The online version of this article (10.1186/s13229-018-0187-7) contains supplementary material, which is available to authorized users.

## Background

Autism spectrum disorder (ASD) is a life-long neurodevelopmental disorder, behaviourally defined by impairments in social communication and the presence of repetitive and restricted behaviours and interests [1]. The considerable variability in the quality and severity of symptoms between individuals with ASD is widely recognized [2]. For example, social communicative impairments can be manifested in a range of difficulties in social-emotional reciprocity, in non-verbal communicative behaviours, and in developing and maintaining social relationships [1]. Recognition of this heterogeneity has begun to cast doubt as to whether a truly diagnostic biological or behavioural marker for ASD exists (which differentiates all or the majority of people with ASD from typically developing individuals or those with other neurodevelopmental/psychiatric conditions) and prompted an increasing interest in the identification of *stratification markers* to parse ASD into more homogeneous subgroups [3]. Biomarkers have been defined as “a characteristic that is objectively measured and evaluated as an indication of normal biological processes, pathogenic processes, or pharmacologic responses to a therapeutic intervention” [4]. Here we use the term “biomarker” in a broad sense to refer to measures of any modality, including cognitive/behavioural tests [4]. *Stratification biomarkers* are then characteristics that vary between ASD subgroups and that map onto differences in their symptom presentation, etiology, need for particular treatments, and/or treatment response.

Because the ability to infer other people’s emotions from their facial expressions is critical for many aspects of social communication, deficits in expression recognition have long been suggested to represent a core impairment in ASD [5]. However, over the past three decades, behavioural studies of facial expression recognition in ASD have produced mixed findings, ranging from reports of profound deficits to apparently intact expression recognition skills (see e.g., [6, 7] for recent reviews). A review of this literature suggests that in ASD, the presence and severity of expression recognition deficits on experimental tests is influenced by both participant characteristics—age and ability level—and task requirements [7]. On the whole, deficits in recognizing *basic* emotion expressions appear to be predominantly found in children and low-functioning individuals (i.e., people with ASD who also have intellectual disabilities) [5, 8]. By contrast, high-functioning individuals with ASD (with IQ in the normal range) usually perform well on tests that depict facial expressions in a prototypical manner and that used relatively long presentation times [9–11]. However, some studies reported group-level deficits in recognizing expressions of *complex* emotions (e.g., guilt, defiance) [9, 12], expressions that were presented only briefly [13] or subtle expressions displayed with low intensities [14] using morphs [15, 16]. Furthermore, speed-accuracy trade-offs [17], abnormal gaze fixation patterns [18], and differences in underlying neural processes as measured by EEG [19] and fMRI have also been reported [20, 21]. However, some inconsistencies remain even within the different methodologies and sample types. For example, some studies have found mean group deficits in adult ASD samples with IQ in the normal range on tasks that required labeling basic emotions with unlimited presentation times [20, 22, 23], while others reported no deficits in the recognition of complex emotions [24].

Recently, a formal meta-analysis of 48 papers concluded that there is an emotion recognition difficulty in “autism”, with a mean effect size of Cohen’s *D* = 0.80, yet this was estimated to decrease to around 0.40 after adjusting for publication biases [6]. Hence, it remains unclear whether differences in study findings reflect variability in the nature or sensitivity of the many behavioural tests used to assess expression recognition in ASD and/or differences in the severity of deficits among people with ASD.

To ascertain the value of expression recognition deficits as a candidate diagnostic or stratification biomarker we need, first, sensitive tests that incorporate several real-life characteristics. For example, in daily life, facial expressions are usually more subtly displayed than those depicted in most standard stimuli of “prototypical” facial emotion expressions. People also often reveal their emotions for only a very brief time. The observer is then required to identify the facial emotion expression quickly to react appropriately to the person’s feelings. Second, the field needs to move away from a sole focus on mean between-group differences to better understand the frequency and severity of expression recognition deficits among individuals with ASD. For example, in case-control studies, a significant *p* value (especially in combination with small or medium effect sizes) could either reflect (small) deficits in most cases (say, around 1 SD below the typically developing (TD) mean) or may be driven by a subgroup of individuals with severe deficits. Obviously these two scenarios have profoundly different implications for the potential utility of the test in clinical practice.

Hence, the aim of this study was to investigate the frequency and severity of deficits in the recognition of facial expressions of emotion using a task that is more sensitive and naturalistic than previous tests. The Films Expression Task [25] uses still images captured from movie scenes and combines three elements that appear to be challenging for people with ASD: depiction of naturalistic facial expressions, inclusion of a range of both basic and complex emotions, and brief presentation times.

## Methods

### The Films Expression Task

The Films Expression Task [25] consists of 58 trials. In each trial, participants were first presented with an adjective describing an emotional state (e.g., confident, pleased). They were then briefly shown three images one after the other (500 ms each, with a 500 ms blank screen between images). The images were taken from films made in non-English-speaking countries to decrease the probability that participants had seen them or were familiar with the actors.

Within each trial, images present the same actor or actress, but with different emotional expressions. Participants were asked to indicate, by key press, which of the images best matched the target word. In 14 trials, the target emotion was a basic emotion (happy, angry, sad, afraid, surprised, disgusted). In the remaining 44 trials, the target emotion was complex (e.g., mocking, hurt, disappointed, resentful, see Additional file 1 for the list of target emotions). In trials with both basic and complex target emotions, the foils were selected to be similar to the targets in terms of perceptual features and intensity of the expression (see supplementary information [25]). Basic vs. complex emotion trials were presented interleaved, in a fixed-random order. Participants were instructed to respond as *quickly* and *accurately* as possible.

Immediately before the task, participants were presented with the definitions for each word describing a target emotion (e.g., guilty). They were encouraged to ask questions if they were unfamiliar with or did not understand any of the words. Participants were also allowed to review these definitions at the start of each trial, after presentation of the target word.

Task development and validation of the stimuli is described in [25] (supplementary information). Briefly, in pilot phase 1, three researchers suggested adjectives to describe 122 pictures of facial expressions taken from 18 films. In pilot phase 2, 32 native English speakers rated how well each adjective described the facial expression on a scale from 1 to 5 (1 = “it doesn’t match at all”, 5 = “it matches very well”). Adjectives were only carried forward if they had received at least a mean rating of 3.5 or a median rating of at least 4. Ten of these participants then also rated the intensity of the target expression from 1 to 5 (1 = “not intense” to 5 = “very intense”) as well as the intensity of the expressions intended as foils. Targets and foils were then matched based on their expression intensity. In pilot phase 3, 30 different native English speakers were asked to choose one of two simultaneously presented expressions (the target and a foil) that best matched an adjective. Distractors were only included if participants had selected them less than 30% of the time. A fourth final pilot phase included ten new participants and employed the intended task procedure in which three pictures were presented sequentially, one target and two distractors. Only trials with equal or less than 30% errors were carried forward, which resulted in 58 trials with targets taken from 15 films.

### IQ measures

Verbal, performance, and full-scale IQ were assessed using the Wechsler Abbreviated Scale of Intelligence-2nd edition (WASI-II; Wechsler, [26]) or a four-subtest short-form of the Wechsler Adult Intelligence Scale-Third Edition (WAIS-III; [27]). In both instances, the tests included two verbal subscales (vocabulary, similarities) and two non-verbal subscales (block design, matrix reasoning). Standardized scores from the WASI-II and WAIS are comparable.

### Autism Spectrum Quotient

The Autism Spectrum Quotient (AQ) [28] is a self-report questionnaire to assess whether adults of average intelligence have symptoms associated with autism spectrum disorder. The test consists of 50 statements and participants indicate whether they “definitely agree”, “slightly agree”, “slightly disagree”, or “definitely disagree” with each statement. Approximately half the items are worded to usually elicit an “agree” response from neurotypical individuals and half to usually elicit a “disagree” response. The questions cover five different domains associated with the autism spectrum: social skills, communication skills, imagination, attention to detail, and attention switching/tolerance of change.

### Test-retest reliability of the films expression test

Thirty-one participants took part in a test-retest study to assess the reliability of the Films Expression Test. We aimed to recruit participants with a range of expression recognition abilities to ensure that test-retest reliability on this task was stable at both high and low performance scores. Because we hypothesized that individuals with ASD would have lower accuracy scores than typically developing individuals, four participants were recruited from an autism support group. Hence, 27 participants were typically developing, three had a formal diagnosis of autism, and one was suspected of having autism.

Two participants exceeded the intended maximum retest interval of 6 weeks and did not complete the retest, one participant’s retest data was not available after a technical error. This left 28 participants (20 females and 8 males, mean age = 33.48 years; range = 20–57 years, four from the autism support group). Out of the 28 participants, English was a second language for six but all participants described themselves as fluent English speakers. The retest interval ranged from 14 to 34 days, with an average interval of 18.11 days.

All test-retest participants also completed a two-subtest (vocabulary and matrix reasoning) short-form of the Wechsler Abbreviated Scale of Intelligence (WASI-II; [26]). The mean two-scale IQ was 113.07 (range 87–146).

Test-retest analysis revealed an intra-class correlation coefficient of ICC = .74, indicating acceptable reliability. The mean accuracy score at time 1 was 86.64% (SD = 7.49, range 62.07–98.28%) and at time 2 was also 86.64% (SD = 7.93, range 62.07–94.83%). Individual difference scores, calculated between time 1 and time 2 performance, showed that 17 (60.71%) participants had a difference score less than .5 SDs, 6 (21.43%) were between .5 and 1 SD, and 5 were (17.86%) greater than 1 SD.

### Participants

In the main study, participants consisted of 46 adolescents or adults with ASD (34 male, 12 female) and 53 typically developing (TD) individuals (33 male, 20 female). Eighteen TD participants were tested as part of a previous study [25]. Although the ASD group included a higher proportion of males than the TD group, the difference in the sex ratio between the groups was not statistically significant (*χ*^2^ = 1.5, *p* = .21). Mean age of the ASD group was 30.2 years (SD 9.4, 15–50 years) and of the TD group was 27.5 years (SD 7.8, range 14–55 years). Two participants in the ASD group (15 and 17 years) and two participants in the TD group (15 and 14 years) were younger than 18 years. IQ data was available for 42 out of 46 participants with ASD and all participants in the TD group. Mean verbal and full-scale IQ in the ASD group were 113.9 (range 85–140) and 116.0 (range 87–135), respectively, and in the TD group were 114.0 (range 74–146) and 115.5 (range 85–143). The groups did not significantly differ from each other in terms of age (*t*(86) = 2.3, *p* = .12), full-scale IQ (*t*(86) = .02, *p* = .87), verbal IQ (*t*(86) = .0004, *p* = .98), or performance IQ (*t*(86) = .002, *p* = .96). The four individuals with ASD for whom no IQ data were available had attended mainstream schools, which suggests that their IQ was within the normal range.

All participants with ASD except one were native English speakers. In the TD group, 37 of 53 participants were native English speakers. All TD participants for whom English was not their first language described their current English language level as “fluent”. Fourteen individuals performed within 1 SD of the TD mean or above, one individual below 1 SD, and two individual below 2 SDs of the TD mean. Of these two TD participants, one had an above-average verbal IQ (VIQ) (124), indicative of adequate language comprehension and was therefore not excluded. The other TD participant had an accuracy score of more than 4 SDs below the TD mean (male, 32 years, VIQ = 92, first language Uzbek) and was excluded from the analyses reported below. This left 46 people with ASD and 52 people with TD. The study was approved by the South London and Maudsley NHS Trust ethics committee and the UCL Research Ethics Committee. All participants or a parent in the case of participants who were minors gave informed written consent before study participation.

## Results

### Case-control comparisons

**Fig. 1:**
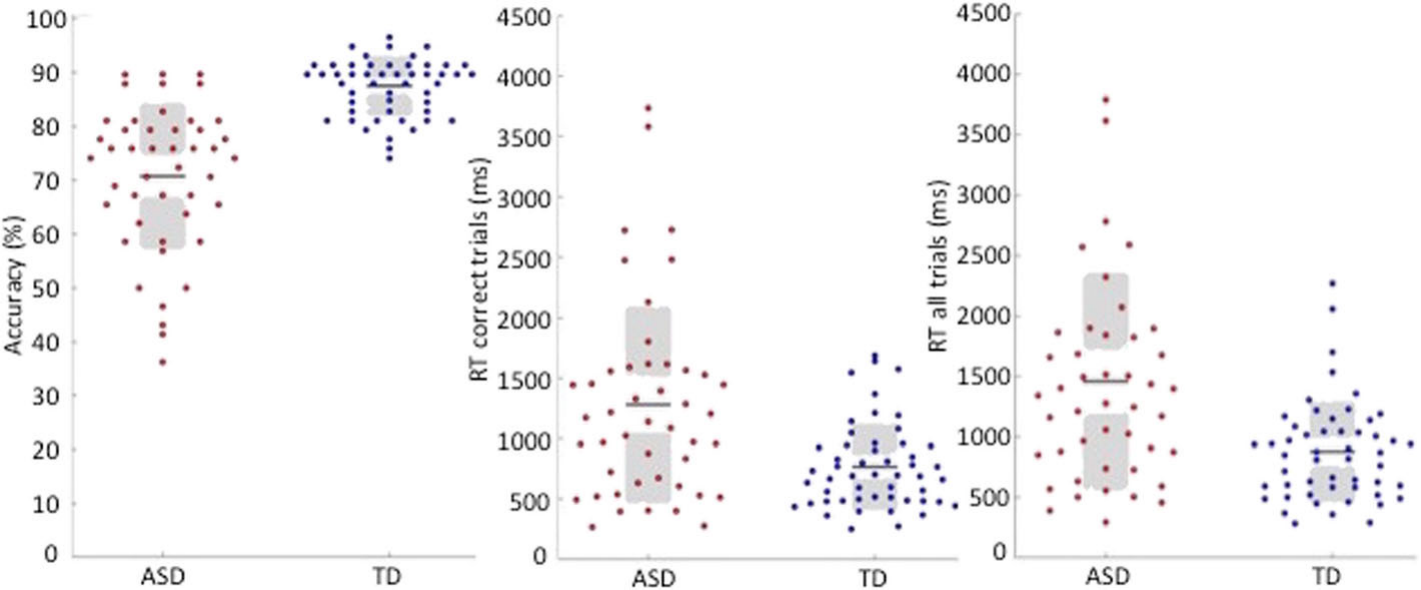
**a** Accuracy: percentage of correct trials, by group. **b** Mean reaction time of correct trials and mean reaction time overall, by group

Descriptive statistics are provided in Additional file 2. The ASD group accurately identified an average of 70.8% (SD = 13.5) of the briefly presented emotion expressions, compared to 87.5% (SD = 5.5) in the control group (see Fig. 1a). The Welch test, which does not assume equal variances, showed that this mean group difference was highly significant (*t*(58.1) = − 7.8, *p* = 1.1 × 10^−10^). The effect size (Cohen’s *d*) was 1.62. We used bootstrapping in Matlab version 8.3.0.532 (Mathworks, Natick, MA) to estimate the 95% confidence interval around this point estimate. We drew 10,000 resamples from the original sample using random sampling with replacement. We obtained a distribution of effect sizes for all the resamples, and using the percentile method (i.e. using the 2.5 and 97.5 percentiles as lower and upper bounds of the CI), the 95% CI was [1.30 2.05].

Analyses of *reaction times* (RT) revealed that the ASD group also responded significantly more slowly (*M* = 1459 ms, *SD* = 899) than the TD group (*M* = 878 ms, *SD* = 414) across all trials (*t*(61.5) *=* 4.02, *p* = .00015, Cohen’s *d* = .83, 95% CI [0.49 1.19]) as well as on their correct trials (ASD: *M* = 1279 ms, *SD* = 812; TD: *M* = 767 ms, *SD* = 352) (*t*(df = 59.7) *=* 3.96, *p* = .0002, Cohen’s *d* = 0.82, 95% CI [0.49 1.17]). These findings remain unchanged when adolescents below the age of 18 years were excluded (accuracy: ASD = 70.2%, TD = 87.7%; *t*(53.5) = 7.9, *p* = 1.4 × 10^−10^) (see Fig. 1b).

### Sex differences

Given previous reports of sex differences on several social-cognitive tasks in the typical population and in ASD [29], we performed 2 (group) × 2 (sex) ANOVAs to test whether accuracy, overall RT, or RT on correct trials differed between males and females overall or in either group. These tests confirmed the significant effects of group on accuracy (*F*(1,97) = 65.0, *p* = 2.3 × 10^−12^) and RTs (all *p*s < .00007) but there were no significant effects of sex (all *p*s > .5)) or group by sex interactions (all *p*s > .11). This finding indicates that the overall case-control difference cannot be attributed to differences in the slightly higher male:female ratio in the ASD than those in the control group.

### Basic vs. complex emotions

Next, we explored whether in the ASD group, the expression recognition impairments reported above were driven by specific problems in identifying complex emotional expressions. To do so, we separately analysed trials with simple vs. complex target emotions (see Additional file 1).

**Fig. 2:**
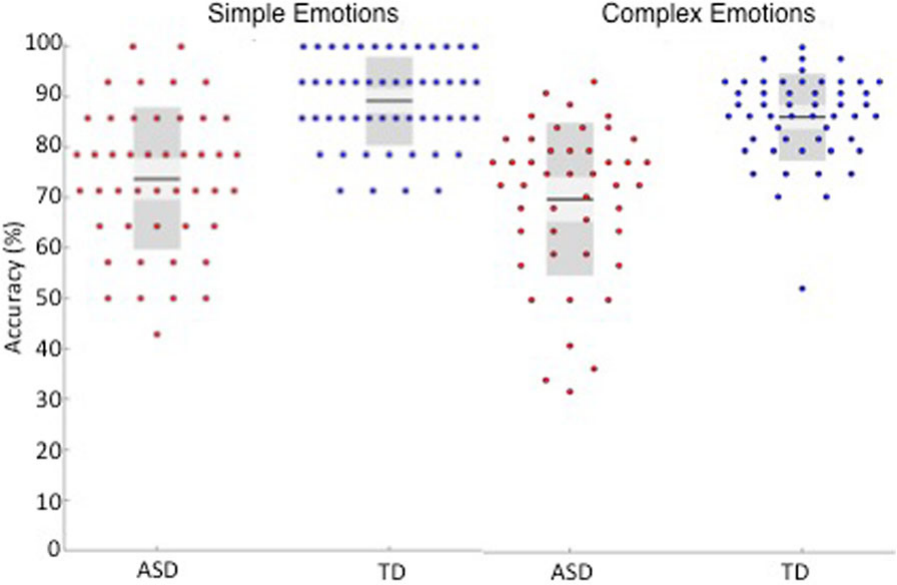
Percentage of correct trials, by emotion category and group

A 2 (group) × 2 (emotion category) repeated-measures ANOVA revealed the above reported significant main effect of group (*F*(1,96) = 70.7, *p* = 3.8 × 10^−13^) and a significant effect of emotion category (*F*(1,96) = 5.9, *p* = .016) on response accuracy, such that on average, both groups were better at recognizing simple than complex emotions. The group x emotion category interaction was not significant *F*(1,96) = .14, *p* = .7) (see Fig. 2).

### Correlations between accuracy and RT scores with age and IQ

Next, we tested whether age, verbal, performance, or full-scale IQ were related to accuracy or reaction time (RT overall or on correct trials) on the Films Expressions Test. In the ASD group, we found no significant relationships between expression recognition variables and age or IQ (all *p* > .09). In the TD group, there was a marginally significant relationship between accuracy scores and FIQ (*r*(52) = .26, *p* = .057), such that participants with higher intelligence also had higher expression recognition scores.

### Frequency and severity of expression recognition deficits in ASD

**Fig. 3:**
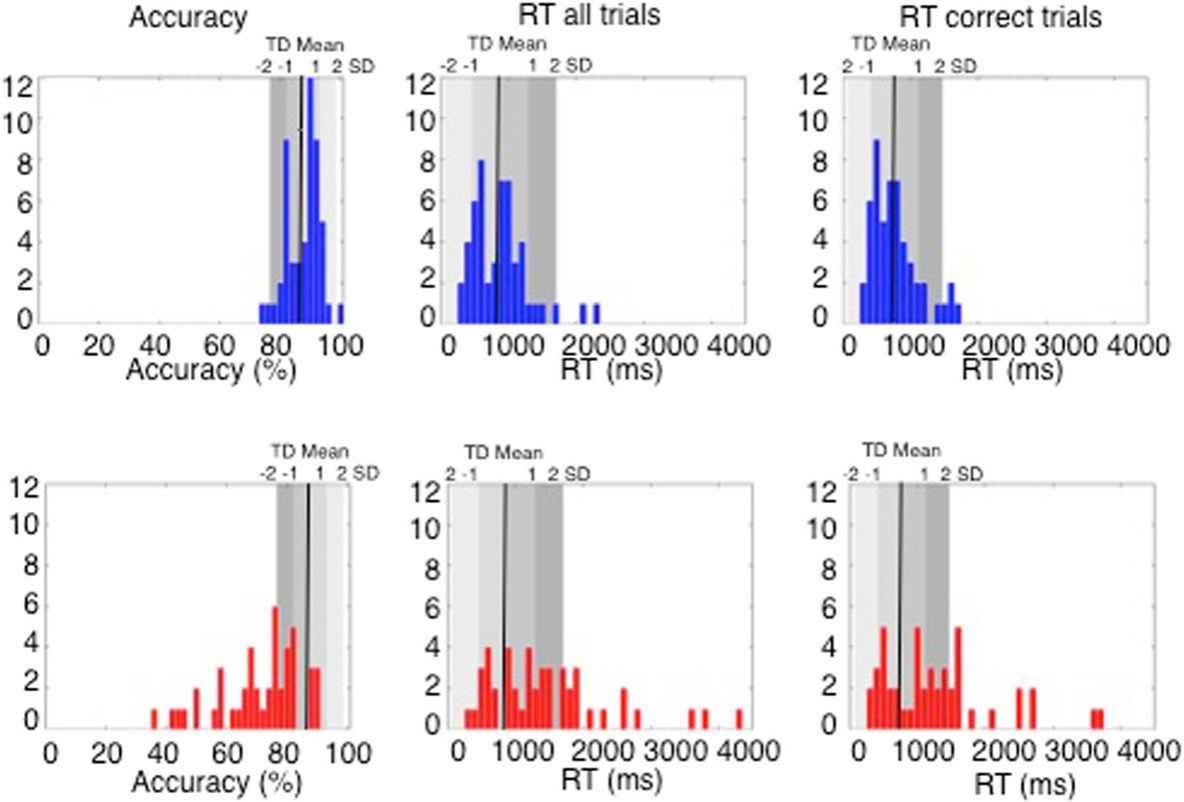
Upper panel: Distribution of TD performance on percentage of accuracy. Lower panel: Distribution ofASD performance on percentage of accuracy. Grey bars denote − 2 to + 2 SDs of the TD means on percentage of correct expression recognition

**Fig. 4:**
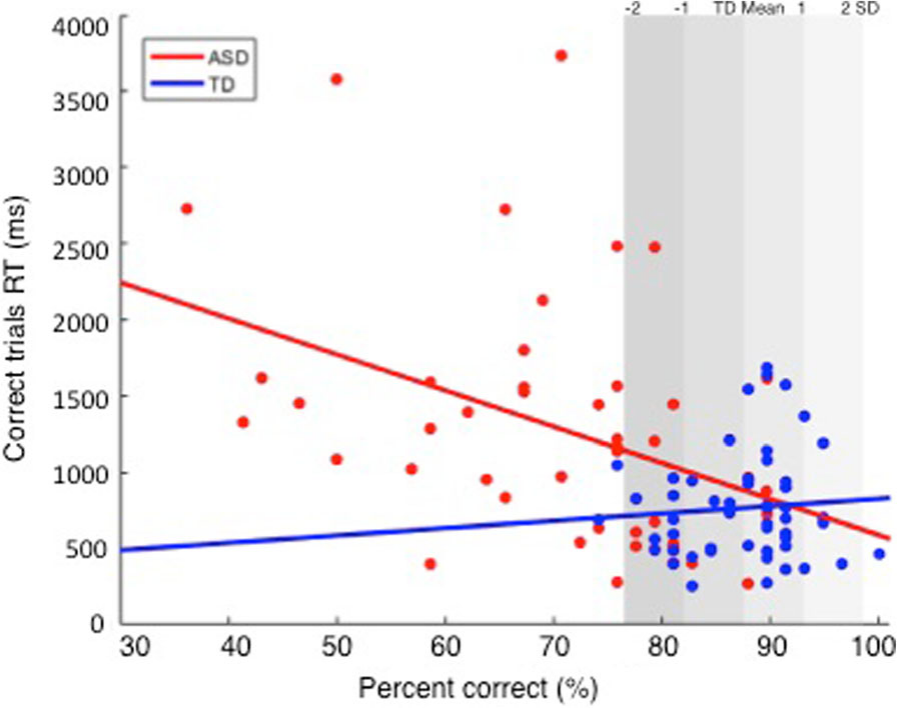
Scatterplot showing percentage of accuracy and RT on correct trials, by group. Grey bars denote − 2to + 2 SDs of the TD means on percentage of correct expression recognition

To establish the frequency and severity of expression recognition deficits in the ASD group, we calculated how far the accuracy score for each individual with ASD deviated from the TD group mean. Analyses of the scores showed that 63% of people with ASD performed more than 2 SDs below the TD mean. Of those, 23.9% performed between 2 and 3 SDs below the TD mean and 39.1% performed more than 3 SDs below the TD mean (see Fig. 3). 21.7% of people with ASD performed between 1 to 2 SDs below the TD mean while 15.3% had accuracy scores within the TD range. The 17-year-old with ASD performed within the 1 SD of the TD range, while the 15-year-old with ASD performed between 2 and 3 SDs below the TD range. As shown in Fig. 4, in the ASD group, accuracy and RT scores were negatively correlated such that individuals with ASD who had higher accuracy scores were also able to correctly recognize the emotion faster than those with low accuracy scores. Therefore, there was no evidence of speed-accuracy trade-off for most of the ASD participants who performed accurately on the task. These results suggest that the majority (63%) of individuals with ASD had severe deficits, while a small subgroup of around 15% of people with ASD showed intact expression recognition skills on this test.

### Correlations between accuracy and RT scores and ASD symptoms

**Fig. 5:**
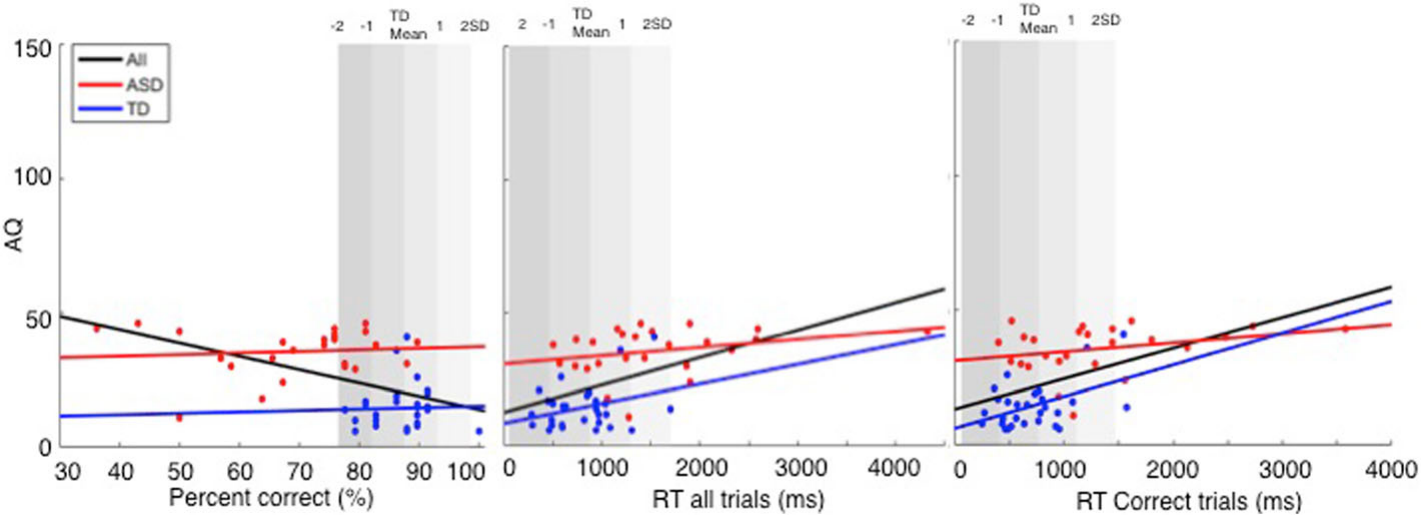
Scatterplot showing the relationship between AQ scores and **a** accuracy scores, **b** overall RTs, and c RTs for correct responses. Regression lines are plotted overall, and for the ASD and TD groups separately. Grey bars denote − 2 to + 2 SDs of the TD means on percentage of correct expression recognition, RTs, and RTs on correct trials

Finally, we investigated correlations with ASD symptoms in a subset of *N* = 53 participants (ASD = 25, TD = 28) for whom AQ data was available (see Fig. 5). When both groups were collapsed, we found a significant negative correlation between the percentage of correct responses on the Films Expression Task and higher ASD symptoms *r*(53) = − .48, *p* = .0002. However, when the ASD and TD groups were considered separately, there were no significant relationships in either group (ASD *r*(25) = .09, *p* = .66, TD *r*(28) = .03, *p* = .88). This suggests that the negative correlation for the overall sample could be explained by the performance differences between the two groups.

A somewhat different picture emerged for RTs. We found that longer reaction times on correct trials were associated with more ASD symptoms. This was significant overall, i.e., when both groups were collapsed (RT_correct trials_: overall *r*(53) = .53, *p* = .00003) and in the TD group (TD *r*(28) = .48, *p* = .012). Although a similar pattern was found in the ASD group, it did not reach statistical significance (ASD *r*(25) = .29, *p* = .16).

Likewise, for overall RTs, we found a significant relationship with ASD symptoms when both groups were collapsed (RT_overall_: overall: *r*(53) = .54, *p* = .000026). When the TD and ASD groups were analysed separately, similar but non-significant patterns were found (TD: *r*(28) = .31, *p* = .10, ASD: (*r*(25) = .29, *p* = .16).

## Discussion

This study investigated the frequency and severity of impairments in facial expression recognition in ASD using a test that captured several features of expression recognition in daily life. A test-retest study showed reliability of .74, which is considered adequate for a test to be of use in clinical settings [30, 31]. At the group level, we found highly significant differences in accuracy and response times between a group of high-functioning adults and adolescents with ASD and an age- and IQ-matched TD control group. Effect sizes of the accuracy scores were more than twice as large as those estimated in a recent meta-analysis of studies on emotion recognition in ASD and more than four times larger than estimates that accounted for publication biases [6].

As a first step towards ascertaining whether deficits in expression recognition may serve as a diagnostic or stratification marker for ASD, we investigated the frequency and severity of these deficits. This revealed that 63% of people with ASD had severe deficits, which would be expected to create substantial social communicative difficulties. For example, participants who performed below 3 SDs of the TD mean failed to identify the briefly displayed target emotion expression in 3–7 out of 10 trials. Translated into real-life settings, it is easy to see how this may impact the ability to, for example, modulate a conversation or to respond empathically [32]. However, we also found considerable variability in that 21.7% performed between 1 to 2 SDs below the TD mean and 15.3% evidenced expression recognition skills indistinguishable from TD individuals (within 1 SD of the TD mean) as indexed by the combination of both accuracy and RT scores. This variability in expression recognition skills among individuals with ASD was unrelated to age or IQ (verbal or non-verbal). However, it should be borne in mind that the IQ range in the current sample was restricted to the normal range, so that this finding may not generalize to individuals with ASD and intellectual disabilities.

The present findings are broadly consistent with other recent efforts to parse heterogeneity in ASD. In particular, Lombardo and colleagues [33] used unsupervised hierarchical clustering approaches to identify ASD subgroups on the basis of item-level performance on the Reading the Mind in the Eyes Test (RMET).The RMET is a widely used mentalising task that also involves a strong emotion recognition component [34] as it requires participants to identify the mental or emotional state of a person from their eye region only. The study (which comprised a discovery and replication cohort) identified five ASD subgroups and four TD subgroups. For individuals with ASD, 45–62% showed what the authors termed “clear to immediate impairments”, while two subgroups (19–36%) performed more than 2 and up to 11 SDs below the TD means - depending on the TD subgroup that was used as comparison. It would be valuable for future studies to directly compare performance on the RMET and Films Expression Tasks or combine information from both measures to derive a composite score.

A second requirement for a quantitative stratification marker is to demonstrate clinical relevance, for example, that people who fall within a certain range of deficits differ from those without abnormalities (normal range) in terms of symptom severity. To begin to address this, we conducted correlation analyses between severity of expression recognition deficits and ASD symptom severity, as measured by the AQ. When the ASD and TD groups were combined, we found both lower accuracy in expression recognition and higher RTs to be moderately related to greater severity of autism traits. However, whereas accuracy scores were not sensitive to the severity of ASD symptoms within the ASD and TD groups, respectively, longer reaction times on correct trials were significantly related to greater ASD symptoms in the TD group. A similar relationship was found in the ASD group with moderate correlation coefficients which, however, did not reach statistical significance. These correlation analyses should be viewed as preliminary, because AQ scores were only available in about half of the participants. As a consequence, the sample sizes were small when split by group, which reduced our power to detect a significant effect. In addition, the AQ is a composite measure of a range of autism-related traits, including features such as intolerance of changes that may not be expected to relate to expression recognition. This may have diluted potentially higher associations with more specific social communicative impairments.

In sum, our findings revealed that the majority of individuals with ASD had severe deficits in expression recognition deficits but also that a sub-sample of individuals with ASD had no behavioural impairments on this task. This indicates that expression recognition may more likely serve as a stratification as opposed to diagnostic marker, subject to further substantiation that subgroups with/without expression recognition deficits also differ in symptom severity or adaptive behavior. One implication of the current findings is that intervention programmes that specifically target expression recognition may be valuable for a substantial proportion of people with this disorder.

### Study limitations and future directions

Several potential limitations of the current study as well as implications for future research should be considered. First, we deliberately chose a task that incorporated several factors crucial for expression recognition in real-life situations, including a wide range of simple and complex target emotions, and relatively short presentation times. As a consequence, could factors unrelated to expression recognition per se have contributed to the deficits observed in some of the ASD participants, for example, comprehension of the target words or other cognitive impairments/ anomalies?

If difficulties in comprehending some of the emotion words accounted for performance impairments, one would expect larger group differences in trials with complex target emotions than basic emotions. However, separate analyses, split by emotion target (simple vs complex), showed significant impairments in the ASD group on trials with basic as well as complex emotions. Basic emotion words are typically understood by the age of 6 years or earlier. Also most of the complex emotions used here are typically understood by age 8 (see Additional file 1), and the ASD participants had verbal IQs within (and mostly above) the population average. However, recognition of complex emotions also requires an understanding of the mental states they describe [34] and some high-functioning individuals with ASD may also have deficits in theory of mind [35]. Therefore, future studies should probe comprehension of the different emotion words and account for individual differences in understanding particular words and their emotional state—independent from recognition of their manifestation in facial expressions.

**Fig. 6:**
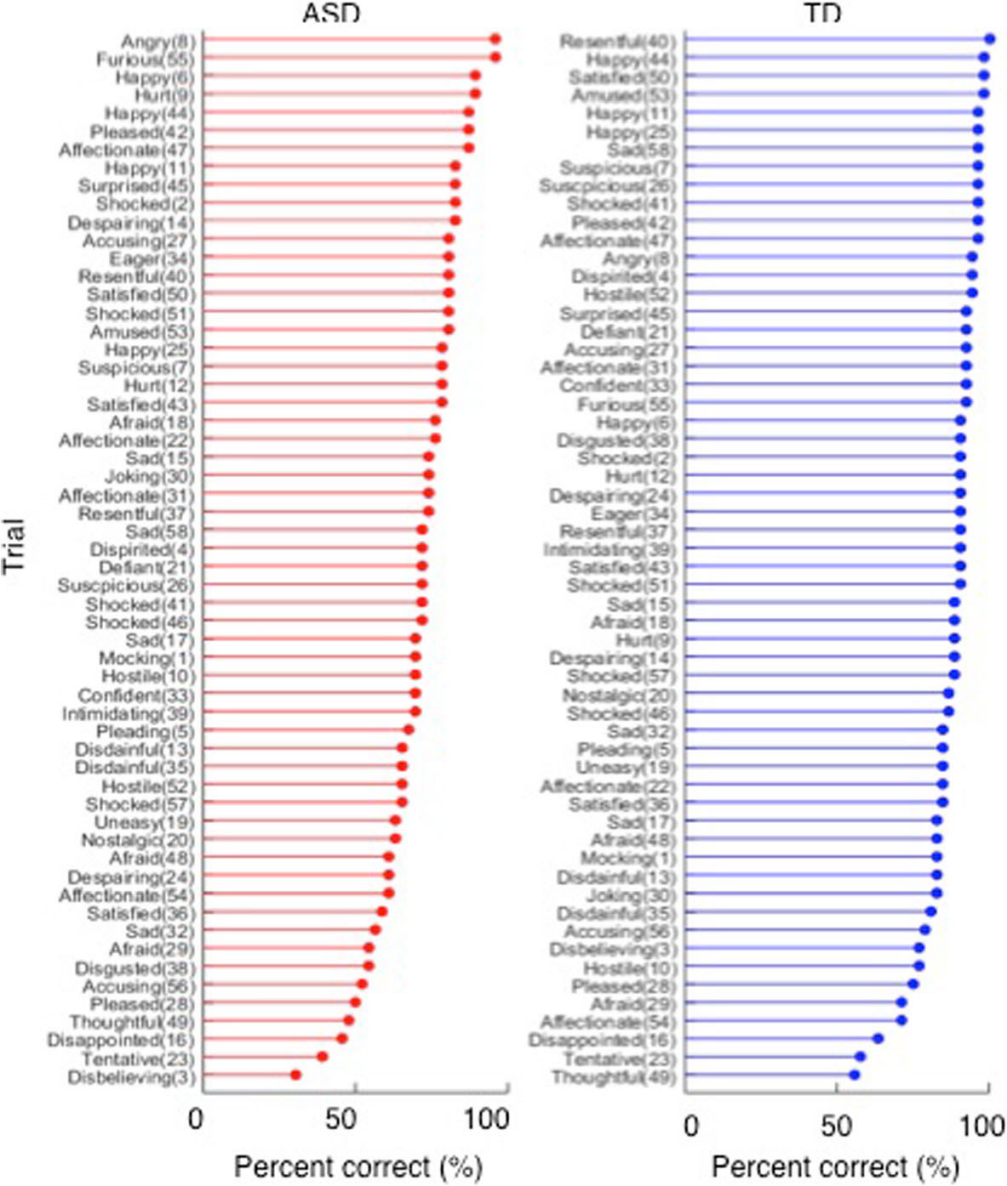
Percentage of correct responses, by trial. **a** ASD group. **b** TD group

Previous studies reported that expression recognition deficits varied with the type of emotion, such that recognition of fear has been found to be more impaired than happiness (see [6] for a review). The naturalistic character of the task did not enable us to systematically analyse recognition of different types of emotion expressions (fear, anger, disappointment, etc.) as 34 different emotion adjectives were included, and many of the target emotions were only used once. However, descriptive trial-by-trial analyses, split by group (see Fig. 6) suggest that the mean percentages of correct response not only varied *between* target emotions but in some instances also *within* target emotions. For example, among the ASD group “angry” and “happy” trials had the highest percentages of correct responses whereas trials with the target emotions “disappointed”, “tentative”, and “disbelieving” had the lowest percentages. For “sad” trials, correct recognition ranged from 56.5 to 73.9%. The latter finding may be due to the fact that the difficulty level of a trial is influenced by the nature of the target emotion and also by the expressions in the distractor stimuli used in a trial.

Could more general problems in rapid visual processing have contributed to deficits in recognizing relatively briefly presented (500 ms) facial expressions of emotions? This alternative explanation seems unlikely for two reasons. Several previous studies reported no deficits in rapid visual processing in ASD [13, 36]. For example, a study that tested recognition of micro-facial expressions, using much faster presentation times (15 and 30 ms) than those employed here reported specific deficits in identifying facial expressions, but not objects in an ASD group [37]. Moreover, as indicated above, our trial-by-trial analyses (see Fig. 6) showed quite variable performance scores between trials in the ASD group, with two trials being correctly identified by over 90% of the ASD participants. This pattern of largely intact expression recognition on some trials but impairments on others would be inconsistent with general problems in rapid visual processing. However, it remains a possibility that other cognitive abnormalities previously reported in (some people with) ASD, such as a detail-focused processing style [38] abnormalities in top-down processing [39] or difficulties with other aspects of face perception [40], may have contributed to behavioural impairments in expression recognition.

The present finding raises the question of what may account for the differences between people with ASD with/without expression recognition deficits. This question cannot be settled here but we discuss potential factors and outline some avenues for future research. A likely possibility is that (some) people with ASD have problems in the neurocognitive mechanisms that represent facial expression information and/or multi-modal expression information. One hypothesis that recently gained prominence is the notion that expression recognition deficits in ASD may be linked to the high frequency of comorbid alexithymia [41, 42], estimated to affect around 50% of people with ASD [43, 44]. Alexithymia is a sub-clinical trait characterized by difficulties in identifying and describing one’s own emotional state [45]. Future work—ideally with larger samples—will be needed to ascertain whether some or all individuals with ASD who exhibit substantial expression recognition deficits also have higher rates of trait alexithymia. Other potential underlying mechanisms may include differences in attention (e.g., to the eye region [11, 46]), or brain structural/functional anomalies [20]. To identify the factors that underpin expression recognition impairments at an individual level will require multi-modal studies and/or designs that assess a range of cognitive functions within each individual. We have included an abridged version of the Films Expression Test and a companion child version in the follow-up assessment battery of the EU-AIMS Longitudinal European Autism Project [47, 48] to test whether the current findings can be replicated in an independent sample more diverse in age and ability level, to further determine the clinical usefulness of our findings, and to investigate potential underlying mechanisms.

## Conclusion

Taken together, our findings show highly significant mean case-control differences in facial expression recognition, with one of the largest effect sizes ever reported in the expression recognition literature of ASD. This suggests that problems with expression recognition are more widespread than currently thought, likely owing to the more naturalistic character of the tasks used here. Nevertheless, we also highlight important variability in expression recognition skills among individuals with this condition and showed that a minority of people with ASD had no behavioural impairments. This finding indicates that the Films Expression Test may serve as a valuable tool to study expression recognition as a candidate stratification marker for ASD.

## Additional files

Additional file 1: Target emotion words, split by age of acquisition (AoA) norms. (DOCX 15 kb)

Additional file 2: Descriptive statistics and contrasts for main variables of interest. (DOCX 19 kb)

## Abbreviations

AQ: Autism Spectrum Quotient
ASD: Autism Spectrum Disorder
IQ: Intelligence Quotient
RT: Reaction time
SD: Standard deviation
TD: Typically developing
WAIS: Wechsler Adult Intelligence Scales
WASI: Wechsler Abbreviated Scales of Intelligence

## References

[1] American Psychiatric Association (2013). Diagnostic and statistical manual of mental disorders.

[2] Charman T. Variability in neurodevelopmental disorders: evidence from autism. In: Riby D, Van Herwegen J, editors. Applied research and key issues in neurodevelopmental disorders. Hove: Routledge Psychology Press; 2015. p. 177–40.

[3] Loth E, Spooren W, Ham LM, Isaac MB, Auriche-Benichou C, Banaschewski T, Baron-Cohen S, Broich K, Bolte S, Bourgeron T (2016). Identification and validation of biomarkers for autism spectrum disorders. Nat Rev Drug Discov.

[4] Biomarkers Definition Working Group (2001). Biomarkers and surrogate endpoints: preferred definitions and conceptual framework. Clin Pharmacol Ther.

[5] Hobson RP (1986). The autistic child's appraisal of expressions of emotion. J Child Psychol Psychiatry.

[6] Uljarevic M, Hamilton A. Recognition of emotions in autism: a formal meta-analysis. J Autism Dev Disord. 2012;43(7):1517-26. 10.1007/s10803-012-1695-5 23114566

[7] Harms MB, Martin A, Wallace GL (2010). Facial emotion recognition in autism spectrum disorders: a review of behavioral and neuroimaging studies. Neuropsychol Rev.

[8] Celani G, Battacchi MW, Arcidiacono L (1999). The understanding of the emotional meaning of facial expressions in people with autism. J Autism Dev Disord.

[9] Baron-Cohen S, Jolliffe T, Mortimore C, Robertson M (1997). Another advanced test of theory of mind: evidence from very high functioning adults with autism or Asperger syndrome. J Child Psychol Psychiatry.

[10] Castelli F (2005). Understanding emotions from standardized facial expressions in autism and normal development. Autism.

[11] Rutherford MD, Towns AM (2008). Scan path differences and similarities during emotion perception in those with and without autism spectrum disorders. J Autism Dev Disord.

[12] Capps L, Yirmiya N, Sigman M (1992). Understanding of simple and complex emotions in non-retarded children with autism. J Child Psychol Psychiatry.

[13] Clark TF, Winkielman P, McIntosh DN (2008). Autism and the extraction of emotion from briefly presented facial expressions: stumbling at the first step of empathy. Emotion.

[14] Wingenbach TSH, Ashwin C (2017). Brosnan: diminished sensitivity and specificity at recognising facial emotion expressions of varying intensity underlie emotion-specific recognition deficits in autism spectrum disorders. Res Autism Spectr Disord.

[15] Kennedy DP, Adolphs R (2012). Perception of emotions from facial expressions in high-functioning adults with autism. Neuropsychologia.

[16] Wang S, Adolphs R (2017). Reduced specificity in emotion judgment in people with autism spectrum disorder. Neuropsychologia.

[17] Sucksmith E, Allison C, Baron-Cohen S, Chakrabarti B, Hoekstra RA (2013). Empathy and emotion recognition in people with autism, first-degree relatives, and controls. Neuropsychologia.

[18] Pelphrey KA, Sasson NJ, Reznick JS, Paul G, Goldman BD, Piven J (2002). Visual scanning of faces in autism. J Autism Dev Disord.

[19] Dawson G, Webb SJ, Carver L, Panagiotides H, McPartland J (2004). Young children with autism show atypical brain responses to fearful versus neutral facial expressions of emotion. Dev Sci.

[20] Ashwin C, Baron-Cohen S, Wheelwright S, O'Riordan M, Bullmore ET (2007). Differential activation of the amygdala and the ‘social brain’ during fearful face-processing in Asperger syndrome. Neuropsychologia.

[21] Wang AT, Dapretto M, Hariri AR, Sigman M, Bookheimer SY (2004). Neural correlates of facial affect processing in children and adolescents with autism spectrum disorder. J Am Acad Child Adolesc Psychiatry.

[22] Macdonald H, Rutter M, Howlin P, Rios P, Le Conteur A, Evered C, Folstein S (1989). Recognition and expression of emotional cues by autistic and normal adults. J Child Psychol Psychiatry.

[23] Boraston Z, Blakemore SJ, Chilvers R, Skuse D (2007). Impaired sadness recognition is linked to social interaction deficit in autism. Neuropsychologia.

[24] Buitelaar JK, van der Wees M, Swaab-Barneveld H, van der Gaag RJ (1999). Theory of mind and emotion-recognition functioning in autistic spectrum disorders and in psychiatric control and normal children. Dev Psychopathol.

[25] Garrido L, Furl N, Draganski B, Weiskopf N, Stevens J, Tan GC, Driver J, Dolan RJ, Duchaine B (2009). Voxel-based morphometry reveals reduced grey matter volume in the temporal cortex of developmental prosopagnosics. Brain.

[26] Wechsler D (2011). Wechsler Abbreviated Scale of Intelligence - Second Edition (WASI-II).

[27] Wechsler D. Wechsler Adult Intelligence Scale. Third ed. London: Pearson; 1997.

[28] Baron-Cohen S, Wheelwright S, Skinner R, Martin J, Clubley E. The autism-spectrum quotient (AQ): evidence from Asperger syndrome/high-functioning autism, males and females, scientists and mathematicians. J Autism Dev Disord. 2001;31:5–17. 10.1023/a:1005653411471 11439754

[29] Lai MC, Lombardo MV, Auyeung B, Chakrabarti B, Baron-Cohen S (2015). Sex/gender differences and autism: setting the scene for future research. J Am Acad Child Adolesc Psychiatry.

[30] George D, Mallery P (2003). SPSS for Windows step by step: a simple guide and reference.

[31] Kaplan R, Saccuzzo D (2001). Psychological testing: principles, applications and issues (5th ed).

[32] Baron-Cohen S (2009). Autism: the empathizing-systemizing (E-S) theory. Ann N Y Acad Sci.

[33] Lombardo MV, Lai M-C, Auyeung B, Holt RJ, Allison C, Smith P, Chakrabarti B, Ruigrok ANV, Suckling J, Bullmore E (2016). Unsupervised data-driven stratification of mentalizing heterogeneity in autism. Sci Rep Oct.

[34] Oakley BF, Brewer R, Bird G, Catmur C (2016). Theory of mind is not theory of emotion: a cautionary note on the reading the mind in the eyes test. J Abnorm Psychol.

[35] Castelli F, Frith C, Happe F, Frith U (2002). Autism, Asperger syndrome and brain mechanisms for the attribution of mental states to animated shapes. Brain.

[36] Rinehart N, Tonge B, Brereton A, Bradshaw J (2010). Attentional blink in young people with high-functioning autism and Asperger’s disorder. Autism.

[37] Wright B, Clarke N, Jordan J, Young AW, Clarke P, Miles J, Nation K, Clarke L, Williams C (2008). Emotion recognition in faces and the use of visual context in young people with high-functioning autism spectrum disorders. Autism.

[38] Happe F, Frith U (2006). The weak coherence account: detail-focused cognitive style in autism spectrum disorders. J Autism Dev Disord.

[39] Loth E, Gómez JC, Happé F (2010). When seeing depends on knowing: adults with autism spectrum conditions show diminished top-down processes in the visual perception of degraded faces but not degraded objects. Neuropsychologia.

[40] Verhallen RJ, Bosten JM, Goodbourn PT, Lawrance-Owen AJ, Bargary G, Mollon JD. General and specific factors in the processing of faces. Vis Res. 2017;141:217-27. 10.1016/j.visres.2016.12.014 28077292

[41] Bird G, Cook R (2013). Mixed emotions: the contribution of alexithymia to the emotional symptoms of autism. Transl Psychiatry.

[42] Cook R, Brewer R, Shah P, Bird G (2013). Alexithymia, not autism, predicts poor recognition of emotional facial expressions. Psychol Sci.

[43] Berthoz S, Haviland MG, Riggs ML, Perdereau F, Bungener C (2005). Assessing alexithymia in French-speaking samples: psychometric properties of the Observer Alexithymia Scale-French translation. Eur Psychiatry.

[44] Lombardo MV, Barnes JL, Wheelwright SJ, Baron-Cohen S (2007). Self-referential cognition and empathy in autism. PLoS One.

[45] Nemiah JC, Freyberger H, Sifneos PE, Hill OW (1976). Alexithymia: a view of the psychosomatic process. Modern trends in psychosomatic medicine.

[46] Klin A, Jones W, Schultz R, Volkmar F, Cohen D (2002). Visual fixation patterns during viewing of naturalistic social situations as predictors of social competence in individuals with autism. Arch Gen Psychiatry.

[47] Loth E, Charman T, Mason L, Tillmann J, Jones EJH, Wooldridge C, Ahmad J, Auyeung B, Brogna C, Ambrosino S, et al. The EU-AIMS Longitudinal European Autism Project (LEAP): design and methodologies to identify and validate stratification biomarkers for autism spectrum disorders. Mol Autism. 2017;8:24. 10.1186/s13229-017-0146-8. 10.1186/s13229-017-0146-8 PMC5481887 28649312

[48] Charman T, Loth E, Tillmann J, Crawley D, Wooldridge C, Goyard D, Ahmad J, Auyeung B, Ambrosino S, Banaschewski T, et al. The EU-AIMS Longitudinal European Autism Project (LEAP): clinical characterisation. Mol Autism. 2017;8:27. 10.1186/s13229-017-0145-9. 10.1186/s13229-017-0145-9 PMC5481972 28649313

