# Supplementary material for: Facial expression recognition as a candidate marker for autism spectrum disorder: how frequent and severe are deficits?
Source: Mol Autism. 2018 Jan 30;9:7. doi: 10.1186/s13229-018-0187-7 (PMC5791186; doi:10.1186/s13229-018-0187-7)
Supplement: Supplementary file 1 — Target emotion words, split by age of acquisition (AoA) norms. (DOCX 15 kb) [file 13229_2018_187_MOESM1_ESM.docx]

**Additional file 1.** Target emotion words, split by age of acquisition (AoA) norms [1]

| **I. Basic emotions (14 trials)** |
| --- |
| Happy |
| Angry |
| Sad |
| Afraid  Surprised  Disgusted |
|  |
| **II. Complex emotions (44 trials)**  **with AoA <8 years (11 trials)** |
| Shocked |
| Hurt |
| disappointed |
| Pleased |
| Furious |
| **AoA >8<10 years (25 trials)** |
| Mocking |
| disbelieving |
| Dispirited |
| Pleading |
| suspicious |
| Hostile |
| despairing |
| Uneasy |
| affectionate |
| Accusing |
| Joking |
| confident |
| Eager |
| Satisfied |
| disgusted |
| thoughtful |
| Amused |
| **AoA >11 years (8 trials)** |
| disdainful |
| nostalgic |
| Defiant |
| tentative |
| resentful |
| intimidating |

1. Kuperman V, Stadthagen-Gonzalez H, Brysbaert M: **Age-of-acquisition ratings for 30,000 English words.** *Behavior Research Methods* 2012, **44:**978-990.
