# Supplementary material for: Facial expression recognition as a candidate marker for autism spectrum disorder: how frequent and severe are deficits?
Source: Mol Autism. 2018 Jan 30;9:7. doi: 10.1186/s13229-018-0187-7 (PMC5791186; doi:10.1186/s13229-018-0187-7)
Supplement: Supplementary file 2 — Descriptive statistics and contrasts for main variables of interest. (DOCX 19 kb) [file 13229_2018_187_MOESM2_ESM.docx]

| Variable | Group | Statistic | Value |
| --- | --- | --- | --- |
| Percentage correct (%) | ASD | Mean | 70.80 |
|  |  | Std. Deviation | 13.51 |
|  |  | Minimum | 36.21 |
|  |  | Maximum | 89.66 |
|  | TD | Mean | 87.51 |
|  |  | Std. Deviation | 5.51 |
|  |  | Minimum | 74.13 |
|  |  | Maximum | 100.00 |
|  | Contrast | *t* | -7.83 |
|  |  | df | 58.12 |
|  |  | *p* | 1.16E^-10^ |
| RT correct trials (ms) | ASD | Mean | 1279.34 |
|  |  | Std. Deviation | 811.94 |
|  |  | Minimum | 268.34 |
|  |  | Maximum | 3732.86 |
|  | TD | Mean | 766.92 |
|  |  | Std. Deviation | 351.69 |
|  |  | Minimum | 252.17 |
|  |  | Maximum | 1685.57 |
|  | Contrast | *t* | 3.96 |
|  |  | df | 59.72 |
|  |  | *p* | 1.99E^-04^ |
| RT all trials (ms) | ASD | Mean | 1459.45 |
|  |  | Std. Deviation | 898.46 |
|  |  | Minimum | 296.80 |
|  |  | Maximum | 4323.82 |
|  | TD | Mean | 878.27 |
|  |  | Std. Deviation | 414.01 |
|  |  | Minimum | 284.67 |
|  |  | Maximum | 2270.89 |
|  | Contrast | *t* | 4.03 |
|  |  | df | 61.58 |
|  |  | *p* | 1.58E^-04^ |
| Simple emotions (proportion correct) | ASD | Mean | 0.74 |
|  |  | Std. Deviation | 0.14 |
|  |  | Minimum | 0.43 |
|  |  | Maximum | 1.00 |
|  | TD | Mean | 0.89 |
|  |  | Std. Deviation | 0.09 |
|  |  | Minimum | 0.71 |
|  |  | Maximum | 1.00 |
|  | Contrast | *t* | -6.50 |
|  |  | df | 74.29 |
|  |  | *p* | 8.22E^-09^ |
| Complex emotions (proportion correct) | ASD | Mean | 0.70 |
|  |  | Std. Deviation | 0.15 |
|  |  | Minimum | 0.32 |
|  |  | Maximum | 0.93 |
|  | TD | Mean | 0.87 |
|  |  | Std. Deviation | 0.07 |
|  |  | Minimum | 0.70 |
|  | Contrast | Maximum | 1.00 |
|  |  | *t* | -6.83 |
|  |  | df | 63.11 |
|  |  | *p* | 3.97E^-09^ |

Additional file 2

Descriptive statistics and contrasts for main variables of interest
